# Dendritic planarity of Purkinje cells is independent of Reelin signaling

**DOI:** 10.1007/s00429-014-0780-2

**Published:** 2014-05-15

**Authors:** Jinkyung Kim, Tae-Ju Park, Namseop Kwon, Dongmyeong Lee, Seunghwan Kim, Yoshiki Kohmura, Tetsuya Ishikawa, Kyong-Tai Kim, Tom Curran, Jung Ho Je

**Affiliations:** 1 X-ray Imaging Center, School of Interdisciplinary Bioscience and Bioengineering, Pohang University of Science and Technology (POSTECH), Pohang, 790-784 South Korea; 2 Department of Pathology and Laboratory Medicine, The Children’s Hospital of Philadelphia Research Institute, Philadelphia, PA 19104 USA; 3 APCTP & IES/NCSL, Department of Physics, Pohang University of Science and Technology (POSTECH), Pohang, 790-784 South Korea; 4 RIKEN SPring-8 Center, 1-1-1 Kouto, Sayo-cho, Sayo-gun, Hyogo 679-5198 Japan; 5 Department of Life Science, Division of Molecular and Life Sciences, Pohang University of Science and Technology (POSTECH), Pohang, 790-784 South Korea; 6 Division of Integrative Biosciences and Biotechnology, Pohang University of Science and Technology (POSTECH), Pohang, 790-784 South Korea; 7 Department of Materials Science and Engineering, Pohang University of Science and Technology (POSTECH), Pohang, 790-784 South Korea

**Keywords:** Dendritic planarity, Purkinje cell, Reelin signaling, Migration, Synchrotron X-ray imaging

## Abstract

The dendritic planarity of Purkinje cells is critical for cerebellar circuit formation. In the absence of Crk and CrkL, the Reelin pathway does not function resulting in partial Purkinje cell migration and defective dendritogenesis. However, the relationships among Purkinje cell migration, dendritic development and Reelin signaling have not been clearly delineated. Here, we use synchrotron X-ray microscopy to obtain 3-D images of Golgi-stained Purkinje cell dendrites. Purkinje cells that failed to migrate completely exhibited conical dendrites with abnormal 3-D arborization and reduced dendritic complexity. Furthermore, their spines were fewer in number with a distorted morphology. In contrast, Purkinje cells that migrated successfully displayed planar dendritic and spine morphologies similar to normal cells, despite reduced dendritic complexity. These results indicate that, during cerebellar formation, Purkinje cells migrate into an environment that supports development of dendritic planarity and spine formation. While Reelin signaling is important for the migration process, it does not make a direct major contribution to dendrite formation.

**Electronic supplementary material:**

The online version of this article (doi:10.1007/s00429-014-0780-2) contains supplementary material, which is available to authorized users.

## Introduction

Planar dendritic arborization is a distinctive morphological feature of mature cerebellar Purkinje cells (PC) (Greg Stuart and Hausser 1999). The planarity of PC dendrites is critical for cerebellar function because planar PC dendrites have optimized structures for connecting with 10^5^ to 10^6^ parallel fibers that traverse along the longitudinal axis of the cerebellum (Kurihara et al. 1997; Ito 2002). Climbing fiber axons from the inferior olive also extend along the dendritic planes, forming synaptic connections with PC (Sugihara et al. 1999). This elegant neuronal architecture is disrupted in the classical mouse neurological mutant *reeler* (Kim et al. 2011) which exhibits ataxia, tremors, imbalance, and a characteristic reeling gait (D’Arcangelo 2006).

In *reeler* mice, most PC fail to migrate to the Purkinje cell plate as a consequence of the absence of the extracellular signaling protein Reelin (D’Arcangelo et al. 1995) and they have poorly developed dendritic arbors lacking dendritic planarity (Kim et al. 2011). It is not known whether the defect in dendritic development is directly caused by the absence of Reelin signaling, regardless of PC migration status, or if it arises as a consequence of the lack of an optimal environment for dendritogenesis in the vicinity of the misplaced PC.

Migration of neuronal precursor cells to their final destinations in the developing brain is crucial for the appropriate formation of neural circuits (Valiente and Marin 2010). Failure in neuronal migration causes functional abnormalities in disorders such as Lissencephaly and Zellweger syndrome (Hatten 1999). Reelin controls neuronal migration by binding to lipoprotein receptors, ApoER2 and VLDR, thus activating tyrosine phosphorylation of Dab1 (D’Arcangelo 2006; Herz and Chen 2006). Crk and CrkL are adaptor proteins that function downstream of Dab1 in the Reelin pathway (Huang et al. 2004; Ballif et al. 2004; Chen et al. 2004). In mice lacking neuronal Crk and CrkL, the cerebral cortex, hippocampus, and cerebellum exhibit impaired migration and dendritogenesis (Park and Curran 2008; Matsuki et al. 2008), similar to those observed in *reeler* mice. However, in contrast to the *reeler* mice in which the cerebellar structure is almost completely disrupted, mice lacking neuronal Crk and CrkL develop rudimentary cerebellar folia. Although approximately 71 % of PC fail to migrate to their final destinations in the cerebellum of mice lacking neuronal Crk and CrkL (Park and Curran 2008), many PC still migrate and align underneath the PC layer. Therefore, this mouse model provides an ideal system for distinguishing the respective contributions of Reelin signaling and local environmental influences to dendritic development. Dendritic development analysis of individual PC requires careful examination of detailed 3-D structures, which is difficult to achieve using conventional 2-D imaging.

Here, we visualized the 3-D microstructure of Golgi-stained cerebella, from mutant and normal mice, at the dendritic level using synchrotron X-ray microscopy (Kim et al. 2011). The distinctive branching patterns and spine morphologies of migrated and non-migrated PC were clearly resolved using this technique. The combined use of phase contrast, based on strongly collimated synchrotron X-rays (Hwu et al. 2002, 2004), and the staining-enhanced absorption contrast produced very high-quality images with limited doses of synchrotron X-rays (Kim et al. 2011). Furthermore, the high-penetration power of synchrotron X-rays (Kim et al. 2012a, b; Margaritondo et al. 2004; Pinzer et al. 2012) made it possible to examine thick specimens with high resolution. This technical approach allowed us to show that the local environment plays a critical role in the development of PC dendritic planarity.

## Materials and methods

### Animals

Generation and maintenance of mice lacking both Crk and CrkL in the brain were previously described (Park et al. 2006; Park and Curran 2008). Although the mutant mice suffer severe growth retardation at 2–4 weeks of age, some of them survived when they were provided with water gel and food at the bottom of the cages. All mouse studies were carried out according to the protocols that were approved by the Institutional Animal Care and Use Committee at the Children’s Hospital of Philadelphia Research Institute.

### Tissue preparation

Two-month-old mice were anesthetized with ketamine (100–300 mg/kg) and xylazine (16–48 mg/kg) and perfused with PBS and 4 % PFA. After perfusion, the cerebella were placed into freshly made 2.5 % potassium dichromate (4-days) and 0.75 % silver nitrate (3-days) solutions at room temperature in the dark (Golgi staining procedure) (Friedland et al. 2006; Golgi 1995). After standard dehydration in ethanol series, the samples were embedded in resin (epon 812 kit: EMS, Hatfield, PA, USA), and each cerebellum was cut into 0.1–5.0 mm slices using a sliding microtome (SM2000R: Leica, Nussloch, Germany). For microtomography, 20 PC from 5 normal and 7 mutant mice were successfully imaged and analyzed. In the case of nano-radiography, 30 PC from 10 normal and 10 mutant mice were also analyzed. For the analyses of all the mutant mice, 2 or 3 PC per each mouse were sampled. Statistical analyses of data were carried out using unpaired two-tailed Student’s *t* test for comparison between two experimental groups. Differences were considered to be significant when probability (*p*) values were <0.05.

### Antibodies

Anti-Crk (22, BD) and anti-CrkL (C-20, Santa Cruz) antibodies, which bind to C-termini of Crk and CrkL, respectively, were used. Affinity-purified rabbit polyclonal antibodies detecting the N-termini of Crk and CrkL are custom-made antibodies generated by Rockland using the following peptides: CPAQPPPGVSPSRLRI-amide, a specific peptide for Crk to generate anti-Crk (75–89), and CPGDYVLSVSENSRVSHYIINS-amide, a conserved peptide in Crk and CrkL for anti-Crk/L (44–64). The numbers in parentheses indicate the first and last amino acid residues in Crk for the peptides. Anti-Calbindin (CB-955) antibody from Sigma was used.

### Immunohistochemistry

For immunohistochemical staining, fixed mouse brains were embedded in paraffin and sectioned at a thickness of 5 μm. Sections were processed through deparaffinization and rehydration followed by antigen retrieval in 10-mM sodium citrate, pH 6.0, for 5 min at 120 °C followed by 10 s at 90 °C using a digital decloaking chamber from Biocare Medical. Slides were blocked in PBS supplemented with 3.5 % bovine serum albumin, and incubated with antibodies overnight at room temperature. Immunoreactivity was detected using biotinylated secondary antibodies and rhodamine- or fluorescein-conjugated avidin (Vector Laboratories). Images of immunostained sections were captured using a Nikon 90i microscope equipped with Roper EZ monochrome and DS-Fi1 color cameras. Images were analyzed using the Nikon NIS-elements software.

### Synchrotron X-ray imaging

The microtomographic experiments were performed with the BL29XU RIKEN Coherent X-ray Optics beamline (SPring-8, Japan: http://www.spring8.or.jp/en) and with the 6D X-ray Micro Imaging beamline (PLS, Korea: http://pal.postech.ac.kr/). The synchrotron X-ray was transported into experimental hutches and the flux was controlled by motorized slits with tungsten blades. A mechanical shutter (Pt–Ir blade) and attenuators (Si) were used to minimize the radiation damage to the sample. Sample was mounted on a high-precision motor-controlled stage with rotational, tilting, and translational resolutions of 0.002°, 0.0009°, and 250 nm, respectively. After passing through the sample, the transmitted X-rays were converted to visible light using a scintillator (CdWO_4_: Nihon Kessho Koogaku Co. Ltd., Hinata Tatebayashi City Gunma, Japan). After being reflected by a mirror and magnified by an optical lens, the image was captured by a charge couple device (CCD) camera. The nano-radiographic experiments were conducted using the 32-ID Transmission X-ray Microscopy beamline (APS, USA: http://www.aps.anl.gov/). Monochromatic X-rays were gathered using a capillary condenser prior to passing through a pinhole and illuminating the mounted sample. The X-rays transmitted through the sample were magnified by a Fresnel zone-plate objective lens and captured by a CCD camera.

### 3-D images acquisition and reconstruction

The microtomographic experiments for the visualization in 3-D geometry were carried out by taking 1,000 microradiographs at every 0.18° rotation step, calibrated with background images. The projected images were reconstructed with the standard filtered back projection reconstruction algorithm using the Octopus 8.5 software (INCT, Zwijnaarde, Belgium). Reconstructed slices consisted of 1,600 × 1,600 pixels in the horizontal and vertical directions. Volume-rendered 3-D images were obtained from the vertically stacked 2-D slices using the Amira 5.2 software (Visage Imaging, San Diego, CA, USA).

### Quantitative analysis

The main criterion to identify PC among various cell types is the typical soma size (approximately 20 μm) in normal and *Crk/CrkL* knockout cerebella: the soma sizes of the other cell types in the cerebella are generally much smaller than 10 μm. To obtain 3-D coordinates of the PC dendrites, reconstructed image stacks of PC were segmented and skeletonized using the Amira 5.2 software. The 3-D coordinates of the PC were automatically analyzed with the Matlab software (Mathworks, Natick, MA, USA). For 3-D Sholl analysis (Fig. 4c; Fig. S1c), a series of concentric spheres of 5 μm increments were drawn around the cell body, and the number of branches between consecutive spheres was counted. For spine analysis, we adjusted the image contrast by altering the display range using the Image-Pro Plus software (Media Cybernetics, Silver Springs, MD, USA). This allowed each spine to be identified. Based on this process, the number of spines per dendritic segment was counted and normalized to 10 μm of dendritic length.

### Fractal dimension

We used the box counting method to measure fractal dimension. First, we obtained skeleton data points of the neurons in 3-D space. We counted the number of boxes [*N*(*r*)] to cover the neurons with the length of boxes (*r*) using a greedy coloring algorithm. A log–log plot of *r* versus *N*(*r*) could be fitted by a straight line with a slope (−*D*), where *D* is the fractal dimension (Fig. S2). A linear least square regression was applied to determine this slope accurately. The appropriate scaling region in the middle was selected to avoid the finite size effect and the limited resolution of the data.

## Results

### Expression of Crk and CrkL in normal and mutant cerebella

**Fig. 1:**
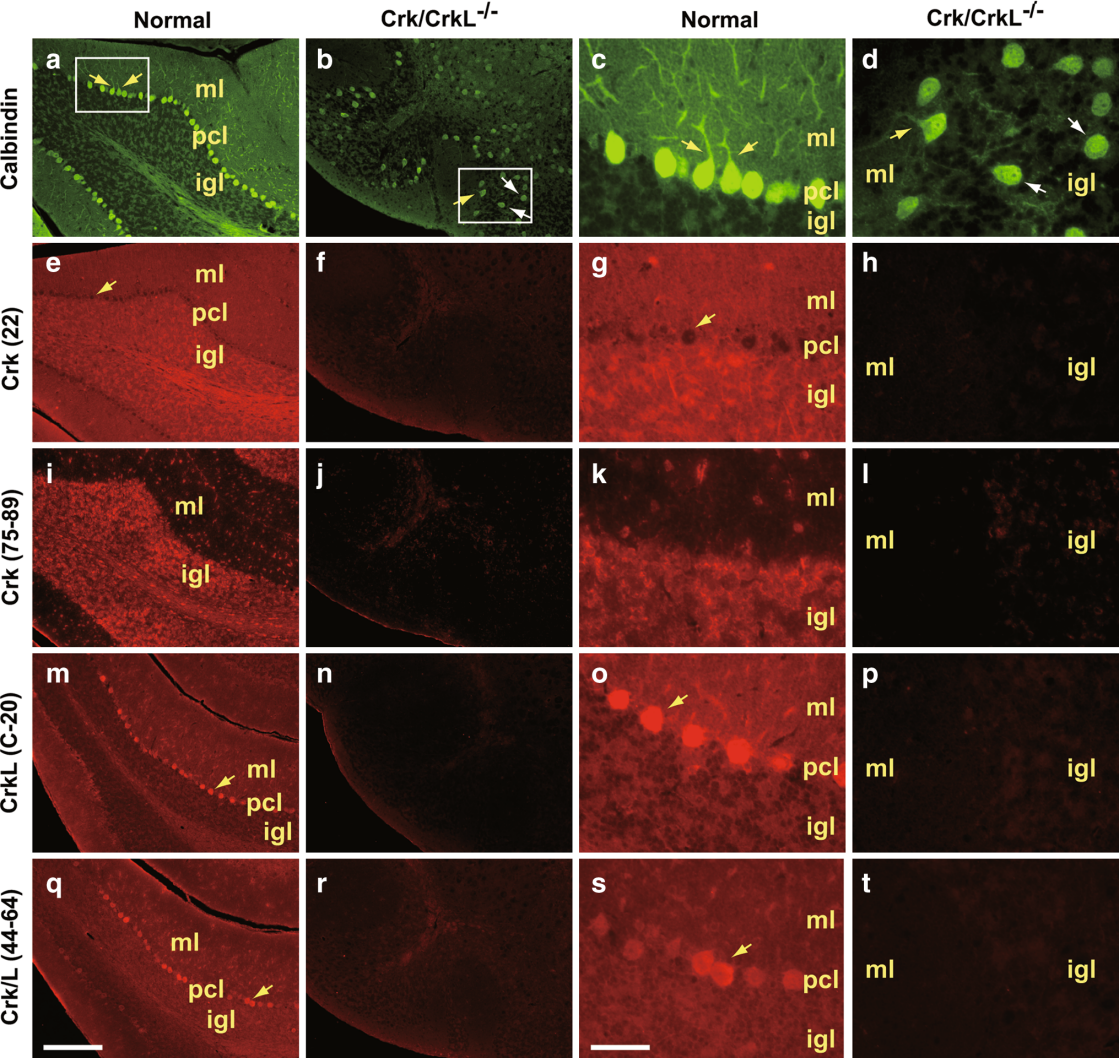
Expression of Crk and CrkL in normal and mutant cerebellum. Calbindin immunostaining of normal (**a**, **c**) and *Crk/CrkL* mutant (**b**, **d**) cerebellum. Panels **c** and **d** are high-magnification images of boxes in panels **a** and **b**, respectively. Crk (22) immunostaining of low (**e**, **f**) and high (**g**, **h**) resolution images of normal and *Crk/CrkL* mutant. Crk (1154) immunostaining of low (**i**, **j**) and high (**k**, **l**) resolution images of normal and *Crk/CrkL* mutant cerebellum. CrkL (C-20) immunostaining of low (**m**, **n**) and high (**o**, **p**) resolution images of normal and *Crk/CrkL* mutant cerebellum. Crk/L (1157) immunostaining of low (**q**, **r**) and high (**s**, **t**) resolution images of normal and *Crk/CrkL* mutant cerebellum. *ml* molecular layer, *pcl* PC layer, *igl* internal granular cell layer

In mice lacking neuronal Crk and CrkL, many PC fail to migrate whereas some PC migrate to the PC plate (Park and Curran 2008). Since *Crk* and *CrkL* genes are deleted by the *Cre* transgene under the Nestin promoter, we examined the possibility that migration of some PC results from incomplete deletion of *Crk* and *CrkL* by Cre. As shown in Fig. 1, PC visualized by staining with Calbindin antibodies align between the molecular layer (ML) and the internal granular cell layer (IGL) in normal cerebellum with their dendritic arbors extended into the ML (yellow arrows in Fig. 1a, c). In mice lacking neuronal Crk and CrkL, most PC fail to migrate and align in the PC plate (white arrows in Fig. 1b, d). However, a subset of PC did migrate and subsequently they developed dendritic arbors (yellow arrows in Fig. 1b, d).

We used several antibodies for Crk and CrkL to visualize the distribution of Crk and CrkL in normal and mutant cerebellum. A mouse monoclonal antibody that binds to the C-terminus of Crk showed widespread staining across the cerebellum except for PC although the IGL was stained stronger (yellow arrows in Fig. 1e, g). A rabbit polyclonal antibody that recognizes the N-terminus of Crk preferentially stained the IGL (Fig. 1i, k). A rabbit polyclonal antibody against the C-terminus of CrkL showed the strongest staining of PC bodies (yellow arrows Fig. 1m, o). A similar staining pattern was obtained using a rabbit polyclonal antibody that recognizes both Crk and CrkL (Fig. 1q, s). Therefore, these observations suggest that although both proteins are widely expressed in the cerebellum, CrkL is enriched in PC bodies, while Crk is enriched in the IGL. In mice lacking neuronal Crk and CrkL, all the Crk and CrkL antibodies tested showed a substantial loss of staining throughout the cerebellum (Fig. 1f, h, j, l, n, p, r, and t). These results indicate that the successful migration of a subset of PC in the mutant mice is not a consequence of incomplete removal of Crk and CrkL proteins.

### 3-D neuronal microstructure of intact cerebellum

**Fig. 2:**
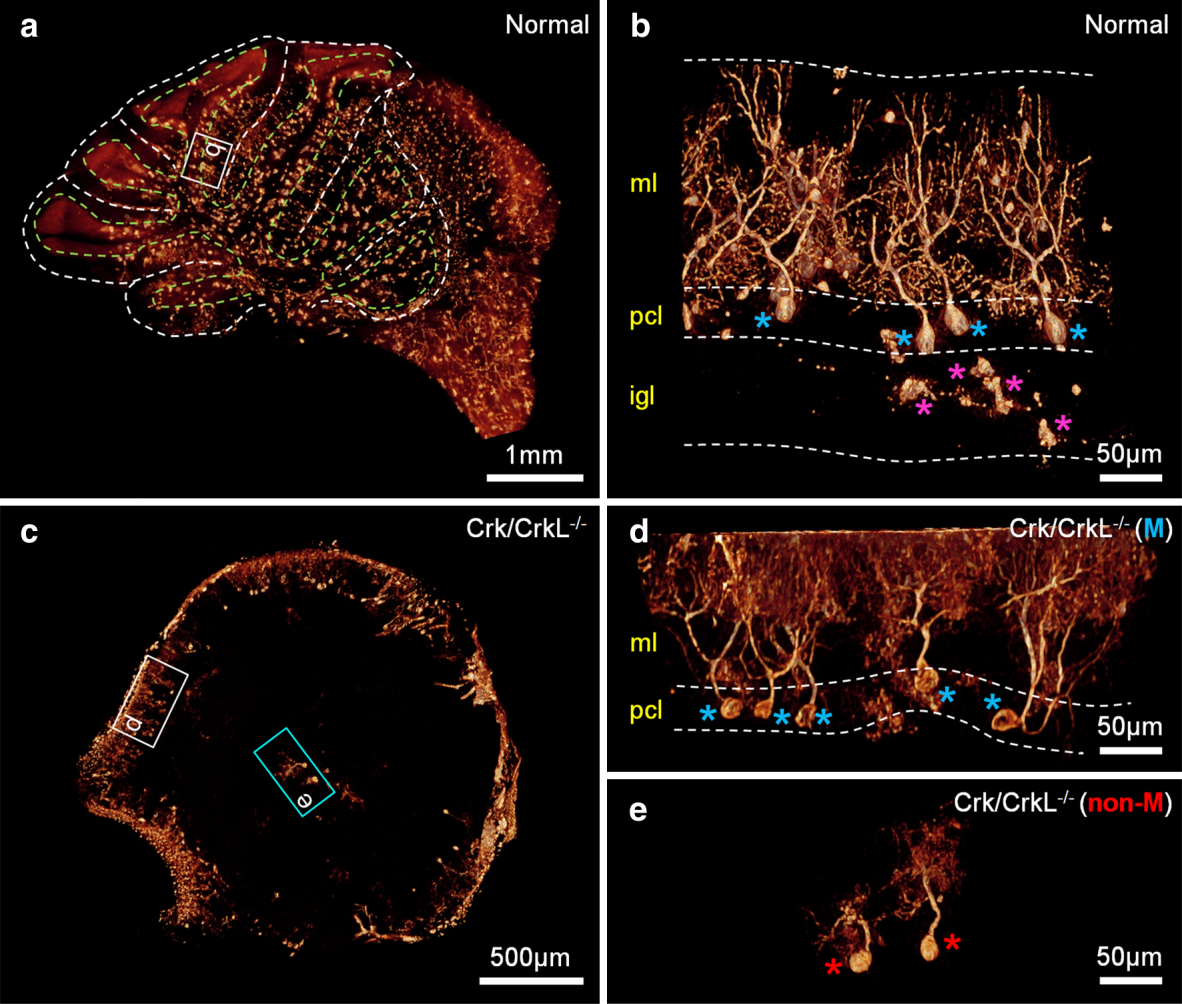
3-D tomographic volume-rendered images of cerebellar tissues. **a** 3-D image of an entire cerebellum in a normal mouse (Movie S1). The lobular and PC layer structures are marked by the *white and light green dashed lines*, respectively. **b** 3-D magnified image of the *white box* region in panel **a** (Movie S2). PC and granule cells are marked by *blue* and *magenta* *asterisks*, respectively. The cerebellar layer arrangement is described by the *white dashed lines*. *ml* molecular layer; *pcl* PC layer, *igl* internal granular cell layer. **c** 3-D image of an entire cerebellum in a *Crk/CrkL* knockout mouse (Movie S3). Migrated and non-migrated PC are marked by the *white and cyan blue boxes*, respectively. **d** 3-D magnified image of the *white box* region in panel **c** (Movie S4). Migrated PC are marked by *blue asterisks*. *ml* molecular layer, *pcl* PC layer. **e** 3-D magnified image of the *cyan blue box* region in panel **c** (Movie S5). Non-migrated PC are marked by *red asterisks*

We took advantage of microtomography of a Golgi-stained cerebellum to reveal its 3-D organization as well as details of PC dendritic arbors (Fig. 2). Figure 2a shows the typical folia (white dashed line) and PC layer (light green dashed line) characteristic of the normal cerebellum (Movie S1). The magnified view in Fig. 2b (boxed area in Fig. 2a) reveals the cerebellar layer arrangement (indicated by the dashed white lines) with PC (blue asterisk) and granule cells (magenta asterisk) (Movie S2). Highly branched PC were aligned in parallel in the PC plate. However, in mice lacking neuronal Crk and CrkL, two classes of PC were identified: those that failed to migrate to their final destinations (cyan blue box), and those that migrated to the PC plate (white box) (Fig. 2c). This is most apparent in the original 3-D image (Movie S3). We noticed that the migrated PC (blue asterisks) exhibited planar structures typical for PC dendrites (Fig. 2d; white box in Fig. 2c; Movie S4), but this was absent from non-migrated PC (red asterisk in Fig. 2e; cyan blue box in Fig. 2c; Movie S5). Thus, the two classes of PC were distinguished both by their location in the cerebellum and by the arrangement of their dendrites.

### 3-D dendritic organization of migrated and non-migrated PC in mice lacking neuronal Crk and CrkL

**Fig. 3:**
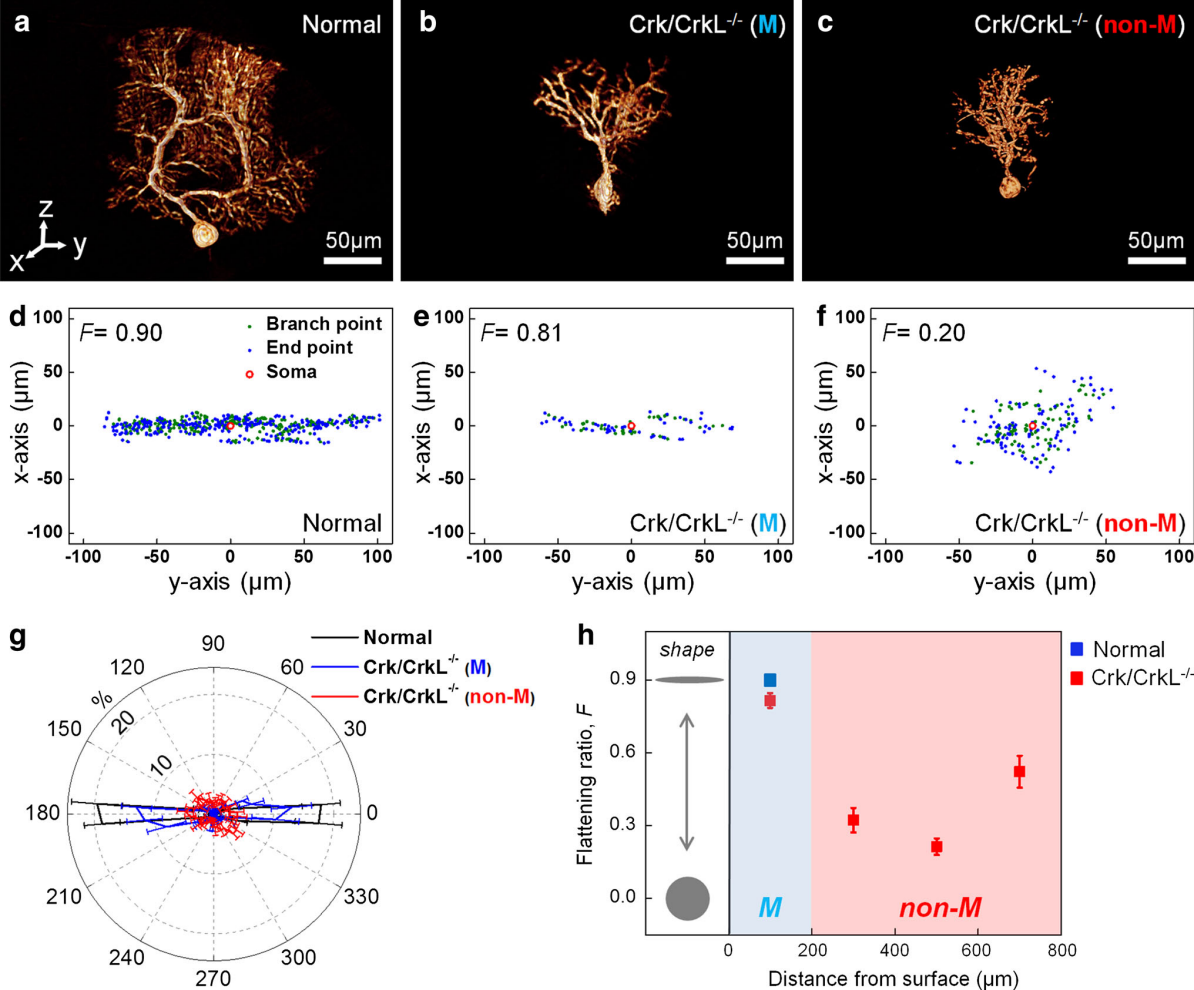
Quantitative characterization of planarity of PC dendrites. **a**–**c** 3-D tomographic volume-rendered image of a normal, a migrated and a non-migrated *Crk/CrkL* knockout PC (Movie S6–8). Here, *y*–*z* defines the sagittal plane, *x*–*z*, the coronal plane, and *x*–*y*, the transverse plane. **d**–**f** Projections on the *x*–*y* plane of the dendritic branch (*green dots*) and the end (*blue dots*) points from the images of panels **a**–**c**, respectively. The soma is marked by a *red circle* at the origin. *F* is the formula for ‘flattening’ ratio, *F* = (*a* − *b*)/*a* (*a* is the length of the semi-major axis in the projections; *b* is its semi-minor axis). **g** Angular distribution in the *x*–*y* plane of the dendritic branch and the end points for 5 normal (*black*), 5 migrated mutant (*blue*), and 10 non-migrated mutant (*red*) PC. The plot shows the percentage of the points found in each 10-degree angular interval. The error bars correspond to the SEM. **h** Flattening ratio of PC projections with the distance from the cerebellar surface (DCS) for 5 normal, 5 migrated mutant (the *light blue* region), and 10 non-migrated mutant (the pink region: *n* = 3 for DCS = 200–400 μm; *n* = 3 for DCS = 400–600 μm; *n* = 4 for DCS = 600–800 μm) PC. The *error bars* correspond to the SEM

The branching patterns of neurons are critical determinants of connectivity and integration (Greg Stuart and Hausser 1999; Mainen and Sejnowski 1996; Javier and Kreitzer 2012). To quantify the branching patterns of PC dendrites in normal and *Crk/CrkL* mutant mice, we examined high-resolution microtomographic images. Figure 3a–c shows 3-D volume-rendered images of PC from normal mice, as well as those representing migrated and non-migrated PC from *Crk/CrkL* mutant mice. The elaborate planar dendritic structure of PC was clearly detected in normal cerebellum using this method (Fig. 3a; Movie S6). Interestingly, migrated PC in *Crk/CrkL* mutant mice also displayed this characteristic planar feature (Fig. 3b; Movie S7); however, in non-migrated PC, dendrites were distributed in a conical array lacking any planar orientation (Fig. 3c; Movie S8). The 3-D volume-rendered images in Fig. 3a–c were used to calculate branch points and dendrite end points, which were projected as green and blue dots, respectively in Fig. 3d–f. In the case of normal PC and migrated PC in *Crk/CrkL* mutant mice, projections aligned along the *y*-axis, indicating a sagittal planar dendritic structure. In contrast, in non-migrated PC, no planar features were evident and dendrites were distributed randomly in the *x*–*y* plane. To quantitate the degree of planarity of the normal, we applied a formula for assessing the ‘flattening’ ratio, *F* = (*a* − *b*)/*a* (*a* is the length of the semi-major axis in the projections; *b* is its semi-minor axis). The flattening ratio was high in migrated mutant PC (*F* = 0.81), similar to that in normal PC (*F* = 0.90), but it was significantly reduced in non-migrated PC (*F* = 0.20).

Next we compared the span, width, and height of PC dendritic arbors as determined by 3-D geometry (Table 1). For mutant arbors, the width in non-migrated cells increased to 75 ± 6 μm, whereas migrated cells had a similar width (25 ± 2 μm) to that observed in normal cells. The arbor span and height were both decreased in mutant PC regardless of whether they had migrated, indicating that Crk/CrkL, and perhaps Reelin signaling, contribute to these features.

**Table 1:** Sizes of PCs, measured for normal, migrated and non-migrated cells in 3-D geometry

	Arbor span (μm)	Width (μm)	Height (μm)
Normal	189 ± 10	18 ± 3	165 ± 6
Crk/CrkL^−/−^ (M)	111 ± 16	25 ± 2	108 ± 17
Crk/CrkL^−/−^ (non-M)	82 ± 7	75 ± 6	94 ± 10

Non-migrated PC exhibited abnormally large arborizations perpendicular to the sagittal plane. This conclusion was confirmed by determining the angular distribution of branch and end points (Fig. 3g); The angular distribution observed in non-migrated mutant cells was essentially random. Therefore, our results suggest that formation of a planar dendritic structure requires migration to the PC plate.

We further examined the correlation between planarity and migration status of PC, as illustrated in Fig. 3h. The distance from the cerebellar surface (DCS) was accurately estimated from 3-D microtomographic images of the entire cerebellum. For the *Crk/CrkL* mutant cells, the flattening ratio of migrated cells in the PC plate (DCS = 0–200 μm; the light blue region) was high (0.82 ± 0.03; red solid square), similar to that observed in normal PC (0.89 ± 0.01; blue solid square in Fig. 3h). However, PC that failed to migrate to their final destination displayed reduced flattening ratios with no significant differences in their migration status: 0.32 ± 0.05 (DCS = 200–400 μm), 0.21 ± 0.03 (DCS = 400–600 μm), and 0.52 ± 0.06 (DCS = 600–800 μm) (red solid squares in the pink region of Fig. 3h). These results indicate that the planarity of PC dendrites requires completion of cell migration but it is independent of the presence of Crk/CrkL.

### Branching rules and fractal dimension of PC revealed by 3-D quantitative analysis

**Fig. 4:**
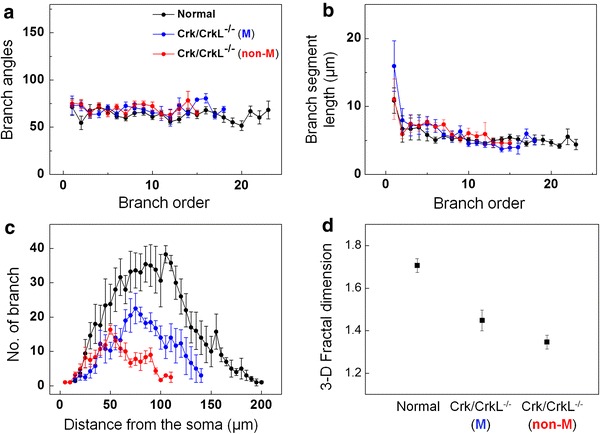
3-D quantitative analysis of the branching rules and the fractal dimension of PC. **a** Branch angles. **b** Branch segment length. **c** Branch numbers, as determined by 3-D Sholl analysis. **d** 3-D Fractal dimension. 5 normal, 5 migrated and 10 non-migrated mutant PC were tested. The *error bars* correspond to the SEM

We measured the branch angles and branch segment lengths of PC in 3-D space (Fig. 4a, b). Dendritic branch angles, measured from the soma to the end of the arbor, were approximately the same in all PC examined: mean value of 66 ± 2° for normal PC, 69 ± 3° for migrated mutant PC and 70 ± 3° for non-migrated mutant PC (Fig. 4a). In addition, branch segment length (the distance from one branch point to the next) for normal PC was constant at 5.5 ± 0.3 μm for all branches except the first branch segment (Fig. 4b). Migrated and non-migrated mutant PC did not show any significant difference in segment length (6.4 ± 0.8 and 6.4 ± 0.4 μm, respectively; Fig. 4b). Thus, our results suggest that Crk and CrkL, as well as Reelin signaling, are not essential for determination of branch angle and the segment length during dendritogenesis.

We next compared the degree of geometric complexity of PC arbors (Sholl 1953) and fractal dimension analysis (Smith et al. 1996) methods (Fig. 4c, d). Both the branch numbers and the fractal dimension of *Crk/CrkL* mutant PC were much smaller than those of normal PC (Fig. 4c, d), indicating reduced geometric complexity. We also observed that the branch number was further reduced in non-migrated PC (Fig. 4c). Consistently, the fractal dimension of non-migrated PC (1.35 ± 0.03) was lower than that of migrated PC (1.45 ± 0.04). These results imply that the geometric complexity is further decreased in non-migrated PC.

### Morphology and density of PC spines

**Fig. 5:**
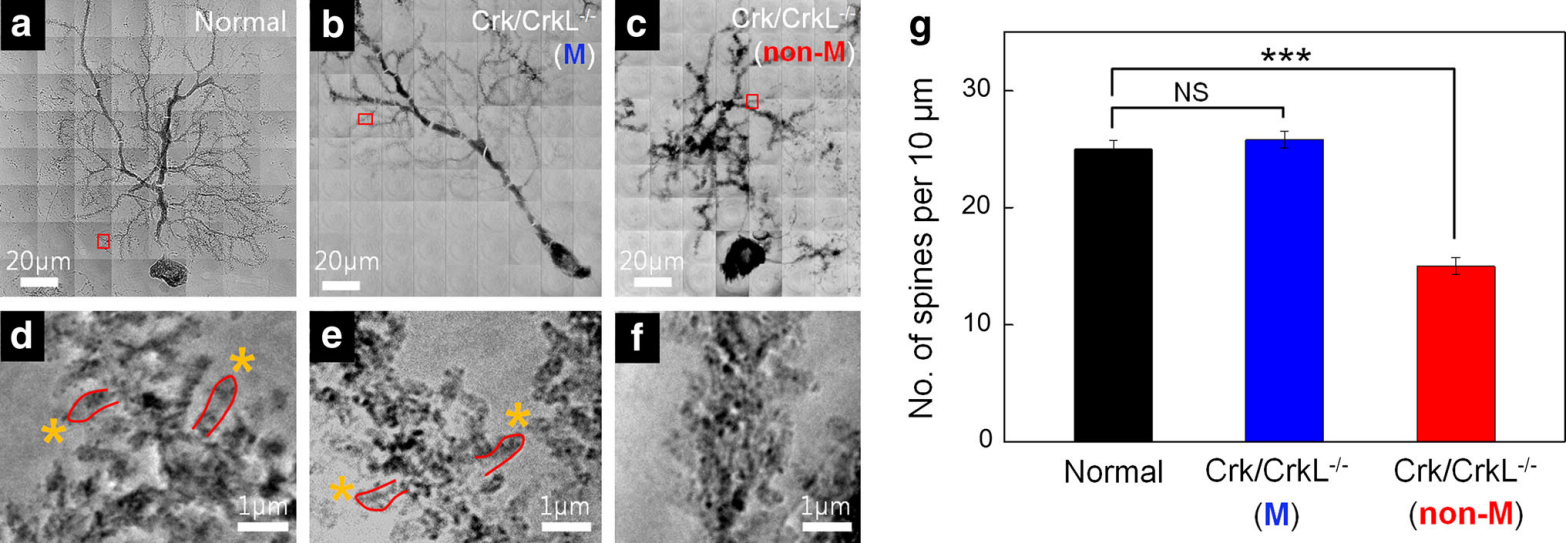
Nano-radiographic images of PC spines and quantitative analysis of their densities. **a**–**c** Entire PC nano-images of a normal, a migrated and a non-migrated *Crk/CrkL* knockout PC. **d**–**f** Magnified images of the box regions in panels **a**–**c**, respectively. **g** Spine numbers per 10 μm of dendrites. Ten mice were analyzed for each group. The *error bars* correspond to the SEM. *NS* not significant; ***p < 0.001 compared to normal

Dendritic spines play important roles in neural information processing and plasticity. The morphology and density of spines have received much attention because of their relationship to their role in synaptic plasticity in the central nervous system (Yuste and Bonhoeffer 2004; Rochefort and Konnerth 2012; Lee et al. 2005). To examine the spines of normal and *Crk/CrkL* mutant PC, we performed nano-radiography (Fig. 5a–c). High resolution (30 nm) and excellent contrast revealed details of spine morphology, as shown in Fig. 5d–f (Chen et al. 2008; Wu et al. 2012). We found that spine morphology was similar in normal (Fig. 5d; box in Fig. 5a) and migrated mutant (Fig. 5e; box in Fig. 5b) PC, but it was substantially distorted in non-migrated mutant PC (Fig. 5f; box in Fig. 5c). More specifically, whereas spines in normal and migrated mutant PC separately sprout from dendrites (yellow asterisks in Fig. 5d, e), spines in non-migrated mutant PC clustered with each other (Fig. 5f). In addition, while the spine density per 10 μm of dendrite was comparable in migrated mutant PC (25.8 ± 0.7) and normal PC (25 ± 0.7), it was significantly decreased (15 ± 0.7) in non-migrated PC (Fig. 5g). The distorted morphology and low density of spines in non-migrated PC may contribute to the reduced functionality of *Crk/CrkL* knockout cerebellum. We propose that migration to the PC plate is essential for the synaptic development and maturation of PC and that the environment in the PC plate contributes to spinogenesis as well as dendritogenesis.

## Discussion

Using 3-D quantitation of dendritic branching patterns, based on a synchrotron X-ray microscopy with Golgi staining, we show that completion of migration is essential for the development of planarity in PC dendrites. Our strategy was based on a combination of factors to improve image contrast and spatial resolution. First, coherence of synchrotron X-rays increased the image quality using the phase-contrast approach (Hwu et al. 2002). Furthermore, heavy metals (potassium dichromate and silver nitrate) used in Golgi staining enhanced image quality by increasing absorption contrast (Kim et al. 2011). A Fresnel zone plate magnifying lens applied to nano-radiography improved the spatial resolution to 30 nm (Chen et al. 2008; Shen et al. 2007). In addition, the high penetration of hard X-rays enabled us to analyze thick specimens, up to several millimeters (Margaritondo et al. 2004; Hwu et al. 2002; Wu et al. 2012). This makes it possible to examine entire cells, multi-cellular structures or tissues without serial sectioning, eliminating a possible loss or distortion of information.

With regard to improvements in the image quality, key issue in our technique is reaching a good compromise between the phase and staining-enhanced absorption contrasts. The two contrast mechanisms are closely related to each other, but there is no general rule to find a compromise. Since the two contrast mechanisms work in different ways for different specimens and for different parts of the same specimens, the only solution is empirical preliminary tests to find the optimum distance between sample and detector and the corresponding optimum level of staining. Meanwhile, there is some room for the improvement in the spatial resolution. The current <30 nm lateral resolution of the zone plate objective is far from the theoretical diffraction limit and also from the estimated instrumentation limits. With even better zone-plate nano-fabrication, the lateral resolution can be further enhanced (Chen et al. 2008, 2011; Lo et al. 2007).

The migration of immature neurons to their final positions and their interactions with neighboring neurons are integral to the development of synaptic circuitry in the brain (Hatten 2002). Studies of neurological mutant mice, such as *reeler*, suggested that, to a large extent, cell positioning is relatively independent of circuit formation, since misplaced neurons seem to be able to make appropriate synaptic connections (Goffinet 1984). However, although this holds at the gross anatomic level, at the fine structure level, this distinction tends to break down as the local environment exerts a profound influence on synaptic maturation. The characterization of mutant mice lacking Crk and CrkL in neurons provided a unique opportunity to distinguish the effects of Reelin signaling on cell migration and PC dendritic arborization.

Here, we demonstrate that environmental differences cause substantial geometric alterations in the branch and spine structures of PC lacking *Crk/CrkL*. Non-migrated PC exhibited conical dendrites (i.e., abnormal 3-D arborization), whereas migrated PC displayed the classic planar dendritic morphology. The altered branching pattern of planar dendrites indicates the importance of extrinsic factors in PC planarity. Further studies will be needed to clarify which extrinsic factors are involved in the formation of the planar dendritic system and how they regulate neural activity in the cerebellar circuit. Second, spines on non-migrated PC exhibited distorted morphologies and they were present at a lower density compared with those on migrated PC. Environmental differences likely affect the ability of non-migrated cells to establish appropriate synaptic connections ultimately resulting in the defects in spinogenesis. Future studies are required to determine which factors are responsible for the deficits in spinogenesis.

Crk family proteins are expressed ubiquitously and they have been proposed to participate in a variety of biological processes, including regulation of cell morphology, migration, proliferation, and differentiation (Feller 2001). In the developing brain, Crk and CrkL were identified as binding partners of tyrosine phosphorylated Dab1 in the Reelin pathway, which is involved in the formation of laminar cortical structures (Rice and Curran 2001; Tissir and Goffinet 2003; Katsuyama and Terashima 2009). The absence of Crk and CrkL from neuronal progenitor cells resulted in structural defects similar to those observed in *reeler* (Park and Curran 2008). We previously reported defective branching patterns of *reeler* PC (Kim et al. 2011). It is noteworthy to compare PC branching patterns of *Crk/CrkL* mutant mice to those in *reeler*. The altered branching patterns and the conical arborization of non-migrated *Crk/CrkL* mutant PC are very similar to those reported in *reeler* PC (Kim et al. 2011). Quantitative analysis of the branching patterns revealed that the branch angles in *Crk/CrkL* mutant PC were approximately the same as those in *reeler* (Fig. S1a). However, in *reeler* PC, the branch segment length was significantly increased (Fig. S1b), and the number of branches was reduced (Fig. S1c), revealing a significantly reduced geometric complexity in the *reeler* PC. Consistently, the fractal dimension was markedly lower, reflecting the serious functional defects in *reeler* mice. In fact, the behavioral abnormality of *reeler* mice is more severe than that of the *Crk/CrkL* knockout mice. Whereas *reeler* mice are severely ataxic and completely lose their postural balance without being able to walk around, *Crk/CrkL* knockout mice are less ataxic and managed to walk around while dragging their paws and frequently losing their postural balance (Park and Curran 2008; our unpublished observation). These behavioral phenotypes are consistent with the corresponding defects in cerebellar structure. The *Crk/CrkL* knockout cerebellum is smaller than normal mice and hypofoliated, but ~29 % of PC still reach the PC layer (Park and Curran 2008). On the other hand, cerebellar foliation is missing in the *reeler* cerebellum and only 5 % of PC are correctly positioned (Goldowitz et al. 1997).

Overall, the 3-D visualization of PC in the whole cerebellum by synchrotron X-ray imaging with Golgi staining allowed us to quantify altered features of dendrites in 3-D geometry. Our quantitative results suggest that migration of PC to the PC plate is crucial for dendritic planarity and spine formation, indicating a significant role for extrinsic factors during neural development. The 3-D visualization of PC provides new insights into PC development. Further understanding of PC development may elucidate etiologies and predict potential treatment strategies for neuronal migration disorders such as Lissencephaly and Zellweger syndrome (Hatten 1999) as well as in Reelin signaling-involved neurodegenerative diseases such as Alzheimer’s disease (Krstic et al. 2013). In addition, we cannot rule out the possibility that in the absence of Crk and CrkL, PC are randomly placed and some randomly placed PC may find themselves in a microenvironment more conducive to normal arborization and synaptogenesis. Further studies will be needed to clarify which extrinsic factors are involved in the formation of the planar dendritic system and how they regulate neural activity in the cerebellar circuit.

## Electronic supplementary material

Below is the link to the electronic supplementary material.

Supplementary material 1 (DOCX 541 kb)

Supplementary material 2 (MPG 20536 kb)

Supplementary material 3 (MPG 24391 kb)

Supplementary material 4 (MPG 14756 kb)

Supplementary material 5 (MPG 13227 kb)

Supplementary material 6 (MPG 4206 kb)

Supplementary material 7 (MPG 14587 kb)

Supplementary material 8 (MPG 6296 kb)

Supplementary material 9 (MPG 10443 kb)
