# Supplementary material for: Dendritic planarity of Purkinje cells is independent of Reelin signaling
Source: Brain Struct Funct. 2014 May 15;220(4):2263–73. doi: 10.1007/s00429-014-0780-2 (PMC4481330; doi:10.1007/s00429-014-0780-2)
Supplement: Supplementary file 1 — Supplementary material 1 (DOCX 541 kb) [file 429_2014_780_MOESM1_ESM.docx]

Original Article

**Dendritic planarity of Purkinje cells is independent of Reelin signaling**

Jinkyung Kim^aŧ^, Tae-Ju Park^bŧ^, Namseop Kwon^a^, Dongmyeong Lee^c^, Seunghwan Kim^c^, Yoshiki Kohmura^d^, Tetsuya Ishikawa^d^, Kyong-Tai Kim^e,f*^, Tom Curran^b*^, and Jung Ho Je^a,g*^

*^a^X-ray Imaging Center, School of Interdisciplinary Bioscience and Bioengineering, Pohang University of Science and Technology (POSTECH), Pohang, 790-784, South Korea*

*^b^Department of Pathology and Laboratory Medicine, The Children’s Hospital of Philadelphia Research Institute, Philadelphia, PA 19104, USA*

*^c^APCTP & IES/NCSL, Department of Physics, Pohang University of Science and Technology (POSTECH), Pohang, 790-784, South Korea*

*^d^RIKEN SPring-8 Center, 1-1-1 Kouto, Sayo-cho, Sayo, Hyogo, 679-5198, Japan*

*^e^Department of Life Science, Division of Molecular and Life Sciences, Pohang University of Science and Technology (POSTECH), Pohang, 790-784, South Korea*

*^f^Division of Integrative Biosciences and Biotechnology, Pohang University of Science and Technology (POSTECH), Pohang, 790-784, South Korea*

*^g^Department of Materials Science and Engineering, Pohang University of Science and Technology (POSTECH), Pohang, 790-784, South Korea*

^ŧ^These authors contributed equally to this work.

*Correspondence and requests for materials should be addressed to J.H.J. ([jhje@postech.ac.kr](mailto:jhje@postech.ac.kr); +82-54-279-2143/fax +82-54-279-2992), K.T.K. ([ktk@postech.ac.kr](mailto:ktk@postech.ac.kr); +82-54-279-2997/fax +82-54-279-2199) or T.C. ([currant@email.chop.edu](mailto:currant@email.chop.edu);+1- 267-426-2819/fax+1-267-426-2791).

**Keywords**

Dendritic planarity - Purkinje cell - Reelin signaling - Migration - Synchrotron X-ray imaging

**
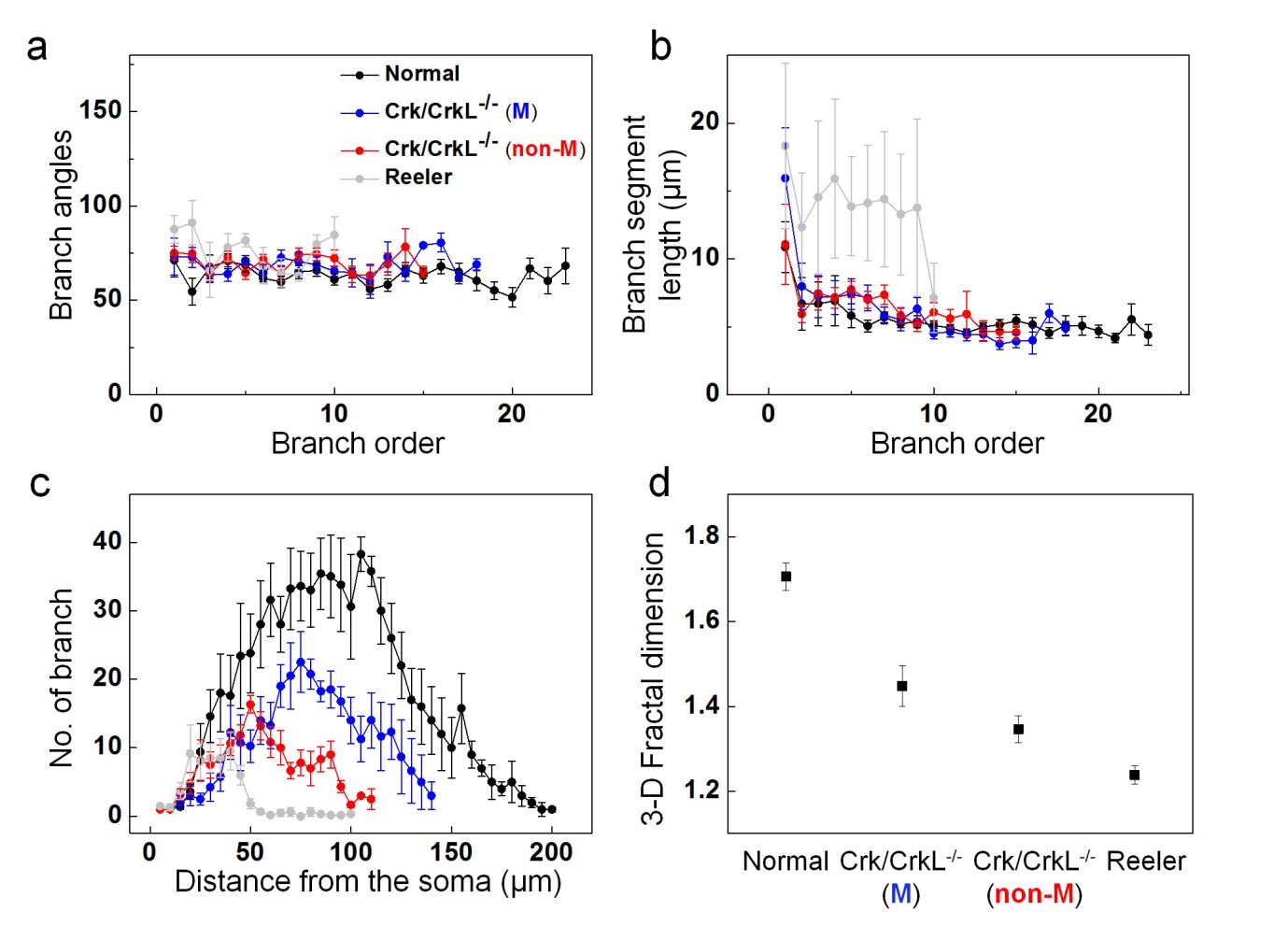
**

**Supplemental figure 1** | **Comparisons of the branching rules and the fractal dimension between *Crk/CrkL* knockout and *reeler* cells.** (a) Branch angles. (b) Branch segment length. (c) Branch number, as determined by 3-D Sholl analysis. (d) 3-D Fractal dimension. 5, 5, 10, and 6 normal, migrated and non-migrated *Crk/CrkL* knockout PC and *reeler* PC were tested, respectively. The error bars correspond to the SEM. The *reeler* data sets are reproduced for comparison purposes from our previous report (Kim, et al. 2011).


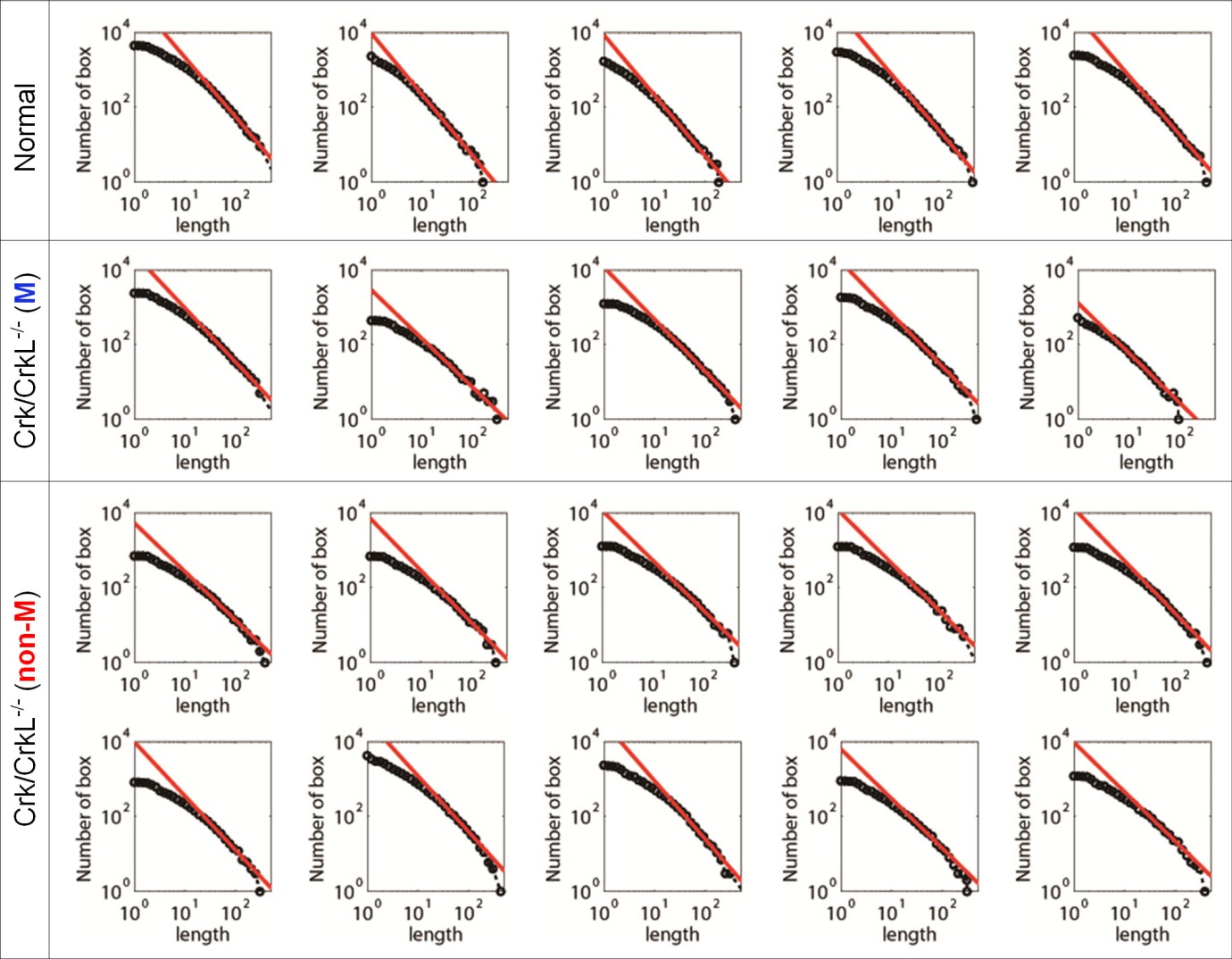


**Supplemental figure 2** | **Box counting analysis of PC.** The graphs show the log-log plots of the length scale and the number of boxes. The slope of the fitting line, in particular the straight segment in the scaling region in the middle, corresponds to the fractal dimension. 5, 5 and 10 normal, migrated and non-migrated mutant PC were tested, respectively.

**Supplemental Movie Legends**

Supplemental Movie 1 |

A microtomographic movie of a thick specimen of a normal cerebellum. The lobular and PC layer characteristic of the normal cerebellum are demonstrated in 3-D geometry.

Supplemental Movie 2 |

A microtomographic movie of the white box region in Fig. 2a. The magnified view reveals the cerebellar layer arrangement with PC and granule cells in 3-D geometry. More specifically, highly branched PC were aligned in parallel in the PC plate.

Supplemental Movie 3 |

A microtomographic movie of a thick specimen of a *Crk/CrkL* knockout cerebellum. Two classes of PC were identified in the 3-D image: those that failed to migrate to their final destinations, and those that migrated to the PC plate.

Supplemental Movie 4 |

A microtomographic movie of the white box region in Fig. 2c. PC that migrated successfully display planar dendritic morphology similar to normal cells of Movie S2.

Supplemental Movie 5 |

A microtomographic movie of the cyan blue box region in Fig. 2c. PC that failed to migrate completely exhibit conical dendrites with abnormal 3-D arborization.

Supplemental Movie 6 |

A microtomographic movie of a normal PC. The elaborate planar dendritic structure of a normal PC is demonstrated in 3-D geometry.

Supplemental Movie 7 |

A microtomographic movie of a migrated *Crk/CrkL* knockout PC. The migrated PC in *Crk/CrkL* mutant mice displays the characteristic planar feature of Movie S6.

Supplemental Movie 8 |

A microtomographic movie of a non-migrated *Crk/CrkL* knockout PC. Dendrites of the non-migrated PC are distributed in a conical array lacking any planar orientation.
